# Protein C Deficiency as a Risk Factor for Stroke in Young Adults: A Review

**DOI:** 10.7759/cureus.7472

**Published:** 2020-03-30

**Authors:** Zainab Majid, Faryal Tahir, Jawad Ahmed, Taha Bin Arif, Anwarul Haq

**Affiliations:** 1 Internal Medicine, Dow University of Health Sciences, Karachi, PAK; 2 Neurology, Baylor University Medical Center, Dallas, USA

**Keywords:** protein c, stroke, thrombophilic disorders, protein c deficiency, anticoagulants, heparin, warfarin, lmwh

## Abstract

Protein C (PC) is a 62-kD vitamin K dependent glycoprotein produced by the liver as a zymogen and is activated by binding to the thrombin-thrombomodulin complex, with protein S (PS) acting as a cofactor. Among its various functions, PC acts as a naturally occurring anticoagulant and its deficiency, either homozygous or heterozygous, predisposes the individual to a state of thrombosis, particularly venous thromboembolism, and mainfests as myocardial infarction (MI), deep venous thrombosis, pulmonary embolism, or stroke. This review discusses the pathophysiology of the anticoagulatory effect of PC, mode of inheritance of its deficiency, the arterial and venous involvement in patients with stroke, and its risk factors. A detailed analysis of published case reports on PC deficiency as a causative agent of stroke in young adults has also been included along with the management of such patients.

## Introduction and background

Protein C (PC), a 62-kD vitamin K dependent glycoprotein, is produced by the liver [1]. It circulates in the blood as an inactive zymogen. When PC is bound to the endothelial proteoglycan thrombomodulin, thrombin catalyzes its conversion into activated PC (APC), i.e., a serine-protease-like enzyme [1]. As a part of its anticoagulant activity, APC causes inactivation of the coagulation factors Va and VIIIa, which are vital for the activation of factor X and the generation of thrombin [1]. The vitamin K-dependent protein S (PS) acts as a cofactor for PC [1]. Besides anticoagulation, APC also exerts anti-inflammatory and cytoprotective functions owing to the endothelial PC receptor (EPCR) and the protease-activated receptor-1 (PAR-1) [1].

The deficiency of PC disturbs the homeostasis between procoagulant and anticoagulant proteins, thereby predisposing the individual to thromboembolism [1]. Due to the reduced velocity of blood flow in the venous circulation, the role of naturally occurring anticoagulants is vital to combat the consequences of prolonged exposure of procoagulant proteins and platelet phospholipids to the vessel wall [1]. It might explain the increased incidence of venous thromboembolism (VTE), i.e., deep venous thrombosis (DVT) and pulmonary embolism at a very young age, in cases of PC deficiency. Although PC deficiency appears to be associated with arterial thromboembolism (ATE) as well, the exact pathophysiology in this regard remains controversial [1].

Heterozygous PC deficiency, an autosomal dominant disorder, exhibits two different types of pathogenic mutations in the long arm of chromosome 2 [1]. Depending on the resultant quantitative deficiency, which is relatively more common, or the qualitative deficiency of PC, these mutations are regarded as type I or type II, respectively [1]. Homozygous or compound heterozygous PC deficiency is relatively rare and can result in neonatal purpura fulminans, intracranial thromboembolism, and thrombosis [1]. Homozygous individuals usually experience death within the first few months of life unless replaced with a PC concentrate in acute episodes and later followed by life-long anticoagulants [2].

The diagnosis of PC deficiency relies on a detailed history of repeated blood clot formation in the patient and the family members and a thorough clinical evaluation, as well as the assays that measure the amount and activity of PC in the body. A range from 0% to 30% is associated with a severe disease, whereas a range of 30% to 70% is a milder variant. The most confirmatory test for PC deficiency is molecular genetic testing (detects mutations in the PROC gene), although it is usually not necessary. This rare condition is most commonly treated with life-long anticoagulant therapy.

Anecdotal cases have reported an association between PC deficiency and ischemic cerebral vessel disease. Nevertheless, a definite association has not yet established or remains controversial [3,4]. In order to identify the etiology in arterial ischemic stroke, testing for inherited thrombophilia is often considered [4]. However, the benefit of screening in this regard has been controversial. Yet, the 2018 American Heart Association/American Stroke Association clinical practice guideline recommends thrombophilia workup in patients with ischemic stroke [4]. Although inherited procoagulant conditions have not been traditionally regarded as risk factors for arterial thrombosis, there are several mechanisms by which they can lead to arterial ischemic stroke. Firstly, DVT and a subsequent paradoxical embolus through a patent foramen ovale can result in an ischemic stroke. Secondly, an imbalance between procoagulants and anticoagulants in individuals with inherited thrombophilias can unnecessarily aid the formation and progression of atherosclerotic plaques through different mechanisms, such as activation of platelets, endothelial and vascular smooth muscle cell dysregulation, and recruitment of monocytes and macrophages [4]. Keeping in view these mechanisms, a recent meta-analysis has demonstrated an association between multiple inherited thrombophilias and the subsequent risk of developing arterial ischemic stroke in adults [4]. Apart from being rare, the incidence of stroke secondary to PC deficiency has been reported in various case reports discussed further in the review. This review aims to highlight the pathophysiology of PC as an anticoagulant along with its various other functions and a detailed discussion of different types of PC deficiency. It also explains the inter-relation between stroke and PC deficiency in the light of previously reported literature.

## Review

Pathophysiology of protein C as an anticoagulant

Anticoagulation was one of the initial roles of PC described in 1960. Since then, the essential mechanism of PC for protection against bleeding and homeostasis has been thoroughly described.

After the exposure of the subendothelial space with blood following a traumatic injury to the vessels, the process of homeostasis is initiated. Primary homeostasis involves the aggregation of the platelets to form a plug, aided by the von Willie band factor, which is released from the endothelial cells of blood vessels and bone marrow. Subsequent activation of the platelet takes place due to the release of α- and δ-granules from the platelets [5]. These activated platelets produce thromboxane A2, platelet-activating factor, and serotonin, which facilitates platelet aggregation, temporarily sealing off the vascular defect. Secondary homeostasis follows next with the involvement of coagulation factors through the intrinsic pathway (containing factors I, II, IX, X, XI, and XII) and the extrinsic pathway (containing factors I, II, VIII, X), giving rise to the formation of fibrin mesh, providing integrity to the clot. Factor V, also known as the labile factor, has an important role as a cofactor for factor X, which, in turn, converts prothrombin to thrombin. Another important glycoprotein required for factor X activation is factor VIII which serves as a cofactor for factor IX.

Our blood is equipped with naturally occurring anticoagulants to regulate blood coagulation and include PC, PS, antithrombin III, and tissue factor plasminogen inhibitor. The vitamin K dependent serine protease, PC, acts by binding to the thrombin-thrombomodulin complex on the endothelium and EPCR, with the latter augmenting PC activation [6]. PS functions as a cofactor for APC and is known to increase proximity between the active site of APC and the membrane [6]. Factors Va and VIIIa act as substrates for the APC, which irreversibly inactivates them through proteolytic activity on cleavage sites, thereby inhibiting their procoagulatory effect. Thus, the deficiency of PC renders the body to the abnormal unrestrained clotting effect and manifests as various life-threatening conditions.

Protein C and other effects

Apart from its role as a naturally existing anticoagulant, PC has several other beneficial functions such as cytoprotection, anti-inflammatory, and barrier stabilization, and recent studies have come forward to establish its therapeutic advantages [6-8]. Table 1 summarizes the diverse functioning of PC.

**Table 1:** Various functions of protein C APC, activated protein C; PAR-1, protease-activated receptor-1; NF, nuclear factor; TNF, tumor necrosis factor; MI, myocardial infarction; MGO, methylglyoxal

Function	Mechanism
Barrier protection	APC not only cleaves and activates PAR-1 in the membrane, which promotes endothelial integrity, but also rearranges cytoskeleton and increases expression of tight junction proteins
Anti-inflammatory	APC inhibits leukocyte and neutrophil adhesion to the endothelial surface and suppresses the activation of pro-inflammatory pathways such as NF-κB and TNF-α
Anti-apoptotic	APC increases the expression of anti-apoptotic genes and provides neuroprotective (by decreasing p53 expression), nephroprotective (by inhibiting reperfusion injury, generating reactive oxygen species, and controlling inflammasome activation), and cardioprotective (by decreasing cardiomyocyte death after MI or protection against MGO apoptotic effects) effects
Role in sepsis	Sepsis is a thrombotic state and consumes APC, thus reducing its level. Hence, administering recombinant APC may inhibit the harmful effects of sepsis-like thrombosis and inflammation

Types of protein C deficiency 

The deficiency of PC, affecting around 0.2-0.4% of the general population, occurs with an equal predilection for males and females, particularly around the ages of 30-40 years [5,9]. Discovered in the early 1980s, the deficiency can either be acquired or congenital. Several factors contribute to acquired PC deficiency, including disseminated intravascular coagulation (DIC), sepsis in young individuals, severe liver disease, vitamin K deficiency, and therapy with warfarin. Congenital deficiency occurs due to a mutation in the gene responsible for producing PC, namely the PROC gene, located at chromosome 2(q13-14). Missense and nonsense are the most common types of mutations, which are seen in 72% of the cases, followed by splicing mutation, which is seen in 9.3% individuals [10]. Although there are 230 probable variants of PC mutation that can result in its deficiency, the two main types are homozygous and heterozygous [10]. Heterozygous mutations arise due to the presence of a single defective gene, either inherited in an autosomal dominant fashion or acquired after a new mutation. On the other hand, the presence of two copies of the defective gene results in homozygous mutation, which is relatively rare and observed in consanguineous marriages. This manifests soon after birth with severe pathologies such as neonatal purpura fulminans, DIC in babies and ATE, and VTE in childhood.

Homozygous and heterozygous varieties are further categorized into type 1 and type 2. Type 1 mutation is described as a reduced concentration of PC antigen and activity; hence, it is a quantitative defect. Type 2 is a qualitative defect resulting from dysfunctional PC with normal antigen limit [11]. The types of PC deficiency have been summarized in Table 2.

**Table 2:** Types and laboratory assays of PC deficiency Table adapted from Knoebl [10] PC, protein C

Deficiency		PC antigen	PC activity
Heterozygous	Type I	Decreased	Decreased
	Type II	Normal	Decreased
Homozygous	Type I	Severely decreased	Severely decreased
	Type II	Normal/moderately decreased	Severely decreased

Stroke and protein C deficiency

Stroke is one of the most common neurological disorders and a leading cause of death globally, affecting 400,000 individuals per annum. It is defined as a neurological deficit as a consequence of the sudden cessation of blood supply to the brain cells either due to obstruction in the form of a thrombus or a ruptured vessel. The commonly encountered underlying causes of stroke comprise hypertension, diabetes mellitus, hyperlipidemia, smoking, and valvular heart diseases, among others. In the absence of these risk factors, the suspicion of thrombophilia as a causative factor emerges. In around 4% of cases of cerebral infarction in young adults, a hematological cause is a culprit [12]. A study evaluated the reason for acute ischemic stroke in 60 young adults and found coagulation inhibitors deficiency in 17% of the patients, out of which PC deficiency was observed in three patients [13]. Similar results were observed in a study conducted by Camerlingo et al. who evaluated the cause of non-hemorrhagic stroke in 50 individuals younger than 45 years of age and found PC deficiency to be the trigger in 6% of the cases [14]. However, a recent article published in 2018 found no significant association of isolated PC deficiency with stroke, but there was a relevant association in cardiovascular events with borderline low levels of PC [15]. These findings were supported by a case-control study that assessed the role of PC, PS, antithrombin III, factor V Leiden, and prothrombin deficiencies as risk factors for ischemic stroke and reported low prevalence (0.9-5.2%) of isolated thrombophilias in patients with first ischemic stroke [16]. Hence, there exists a paucity of large studies to further explore these results and take into account the several case reports that have shown a positive association between PC deficiency and stroke.

Although isolated PC deficiency is a rare cause for the development of thromboembolism, either venous or arterial, the addition of other risk factors can further increase the chances of clot formation. These risks have been thoroughly discussed by Houghton in a review published in 2009 and have been summarized in Table 3.

**Table 3:** Increased risk of symptomatic protein C deficiency when coexistent with other risk factors

Risk factors
Oral contraceptive pills
Pregnancy
Puerperium
Travel through air
Surgery
Smoking
Obesity
Trauma

Veins are generally more prone to developing a thrombus, regardless of the nature of clotting abnormality, since the thrombus formation is related to the Virchow's triad, which includes stasis of blood, hypercoagulable state, and endothelial damage. The fact that veins have a wider caliber as compared to the arteries and hold slow-flowing blood contributes to the higher susceptibility. Venous thrombus is observed in 1 out of every 1,000 people each year and commonly develops in patients with a history of surgery, trauma (particularly fractures), obesity, contraceptive, and certain chemotherapeutic drugs, malignancy, lupus, or simply old age. PC deficiency contributes to 2-5% of cases of VTE [18]. Comparatively, the chances of thromboembolism under the setting of PC deficiency are raised in veins rather than arteries, and the association has been established in the literature [19-21]. The dural sinuses and cerebral veins are interlinked with each other; thus, the formation of thrombus in the former can progress to the latter and vice versa.

ATE due to PC deficiency and other inherited coagulopathies, although not as established as VTE, has been discussed in a few studies. A meta-analysis was performed in 2019 exploring 68 case-control and cohort studies in individuals older than 15 years of age. Out of the 68 cases, PC deficiency was reported in 15 studies, and patients with arterial ischemic stroke had a higher association with PC deficiency than the control group [4]. Mahmoodi et al. in their cohort study also analyzed the risk of ATE in the setting of PC, PS, and antithrombin deficiency, as well as the significance of the positive history of VTE as a predisposing factor [22]. Interestingly, the study reported a 5.6 times higher risk of ATE with only PC and PS deficiencies before the age of 55 years, but no relevance was observed between the history of VTE and ensuing ATE. Of the 308 subjects, 39% found positive for thrombophilia reported PC deficiency in this study [22]. In contrary to the data supporting this association, some studies failed to report any significant link. A study evaluating the causes of arterial ischemic strokes in young individuals revealed hematological pathology in merely 5.8% of the cases, out of which PC deficiency was reported in just one case [23]. Hence, eminent data are still required to confirm and establish the significance in ATE for better management and prophylaxis.

Discussion of the case reports

There are insufficient data regarding the extensive statistics of stroke due to PC and PS deficiency, particularly in adults. Hence, we have gathered resourceful information regarding risk factors, clinical manifestations, family history, management, and prognosis of cases from already published literature (Table 4). We included the cases published in the literature in the English language from 1969 till 2020, in adults aged 18 or above, who either had a history or presentation of symptoms of stroke due to solitary PC deficiency or combined thrombophilia.

**Table 4:** PC deficiency manifesting as stroke in adults: summary of reported cases M, male; N/A, not available; F, female; LMWH, low molecular weight heparin; f/b, followed by; ALOC, altered level of consciousness; DAMA, discharge against medical advice; ACE, angiotensin-converting enzyme; MCA, middle cerebral artery; AT III, antithrombin III; LOC, loss of consciousness; PC, protein C

Year	Sex	Age (years)	Clinical features	PC function (%)	Coexisting thrombophilia	Family history (PC deficiency)	Management	Outcome
2001 [24]	M	29	Left hemiparesis	35	None	N/A	Long-term warfarin	N/A
1985 [25]	F	27	Epileptic fits, diffuse headaches	48	None	Positive	Anticoagulant	Recovered
1985 [25]	F	38	Headache, unstable gait	42	None	Positive	N/A	N/A
1985 [25]	F	34	Unresponsive, restless, vomiting	N/A	None	Positive	N/A	N/A
2013 [26]	F	18	Left-sided hemiplegia	51.7	Protein S	Negative	LMWH f/b anticoagulants	Recovered
2016 [27]	F	42	ALOC, difficulty in breathing	53	None	N/A	LMWH f/b anticoagulants	DAMA
2020 [28]	M	33	Right-sided weakness	41.9	Protein S	Positive	Aspirin, clopidogrel	Recovered
2008 [29]	M	31	Right hemiplegia, sensory disturbance, motor aphasia	46.3	None	Positive	LMWH f/b long-term anticoagulants with warfarin	Recovered
1995 [30]	M	30	Dysarthria, left facial dysesthesia	45	None	Positive	Warfarin	Recovered
2015 [31]	M	33	Left-sided hemiparesis	23.1	None	Negative	LMWH f/b long-term warfarin	Recovered
2003 [32]	F	26	Confusion, personality changes	53	None	Negative	Warfarin, β-blockers, ACE inhibitors, aspirin	Recovered
1990 [2]	M	32	Brachiofacial hemiparesis of the right side, global aphasia	36	None	Positive	Heparin, phenprocoumon	Persistant aphaia, epileptic seizures (MCA recanalized)
1992 [33]	F	31	ALOC, abnormal behavior	<5	None	Positive	AT III therapy, LMWH, warfarin f/b ticlopidine	Recovered
2005 [34]	F	54	Right-sided hemiparesis, LOC	48	None	N/A	Systemic heparinization, barbiturate coma therapy	Transferred for further rehabilitation
1992 [35]	F	39	Left hemiparesis, sensory disturbance, dysarthria	38	None	Positive	Warfarin	Recovered
2013 [36]	M	25	Left-sided weakness, slurred speech	38	Protein S	Negative	Heparin f/b long-term warfarin	Recovered
1995 [37]	F	37	Confusion, drowsiness	33	None	Positive	Warfarin, heparin	Recovered
2004 [38]	F	26	Right-sided visual loss	10	Protein S	N/A	Warfarin, heparin	Able to read, persistent hemianopia
2018 [39]	F	35	Right-sided limb weakness, fatigue	57.6	None	N/A	Long-term aspirin	Recurrent thrombosis (heparin preferred)
2013 [40]	M	41	Motor aphasia, dysarthria, right hemiparesis	47	Protein S	N/A	Heparin f/b warfarin	Recovered
1993 [41]	M	46	Right-sided visual disturbance	54	None	Positive	N/A	N/A
1993 [41]	M	61	Right-sided motor weakness, vertigo, vomiting	57	None	Positive	N/A	N/A
2019 [42]	M	25	Right-sided weakness, altered sensorium, abnormal movements	2.4	None	Positive	LMWH f/b acitrom	Recovered
1998 [3]	M	22	Diagnosed cerebral infarct	47	Prothrombin gene mutation	Positive	N/A	N/A
2009 [43]	M	47	Speech disorder, weakness, and numbness of the right extremities, ALOC	35	None	N/A	Anticoagulants	Recovered

PC deficiency affects males and females in equal numbers, as seen by the cases we reviewed. The majority of these patients fall in the age group of the early twenties or thirties. The lowest age reported by a study in 2013 was an 18-year-old female presenting with left-sided hemiplegia secondary to combined PC and PS deficiency [26]. Contrarily, PC deficiency can be diagnosed as late as 61 years of age after multiple episodes of cerebrovascular accident (CVA) according to a case reported by Kazui et al. [41]. Anatomical distribution of weakness varies with the site of stroke. Predominant involvement of the right side of body manifesting as hemiparesis or hemiplegia secondary to PC deficiency has been found in the literature reported to date. In addition, localized involvement of the right visual field has been observed in two females secondary to stroke in the territory of the left posterior cerebral artery [38,41]. A few patients also had additional clinical features such as altered level of the sensorium, speech disorder, motor or global aphasia, dysarthria, and vertigo. The remainder of the PC deficient individuals presented with either left hemiparesis or hemiplegia. Thromboembolic events presenting with atypical clinical manifestations such as epileptic seizures, restlessness, vomiting, and personality changes also had considerably less activity of PC. Furthermore, a significant association between PC deficiency, livedo reticularis, and recurrent thromboembolism has also been highlighted by Weir et al. [37].

Radiological imaging of brain lesions by CT scan, MRI, or cerebral angiography depicts a variable involvement relative to clinical presentation. Left-sided hemiparesis in PC deficient individuals may occur secondary to right frontal, frontoparietal, or frontotemporoparietal lobe infarct [25,31,35,36]. Matsushita et al. reported a case of a 39-year-old woman presenting with left hemiparesis, sensory disturbance, and dysarthria due to occlusion of the anterior branch of the right middle cerebral artery [35]. However, left hemiplegia secondary to protein C/S deficiency may involve extensive territories of the brain, as reported by Sultan and Malik. CT scan brain of this patient exhibited non-hemorrhagic infarction of left temporal lobe with relative loss of volume of the right cerebral hemisphere and compensatory dilatation of right lateral ventricle [26]. The extent of right-sided weakness also varies with the area of cerebral hemispheres infarcted. Acute infarction of the brain territory supplied by the left middle cerebral artery can present as right hemiplegia, right hemiparesis with motor aphasia and dysarthria, or right brachiofacial hemiparesis, depending on the degree of gray or white matter involved [2,29,40]. Stenosis of the left internal carotid artery with acute ischemic changes in the territory of the left middle cerebral artery can also manifest as right-sided weakness with speech disorder and altered level of consciousness [43]. A few studies have also shown the involvement of distinct parts of the brain presenting as right-sided weakness. PC deficiency presenting as an ischemic lesion of left thalamus with bilateral occlusion of the internal carotid arteries was reported by Kikuta et al. [34]. Similarly, a CT scan of a 35-year-old woman with right-sided weakness and fatigue revealed multiple low-density lesions in the cerebellar hemisphere and vermis [40]. Thrombosis of transverse and sigmoid sinus can also present with right-sided weakness and abnormal movements [42].

Inherited thrombophilia should be suspected in any individual with a positive family history of thrombotic disease. PC deficiency is inherited as an autosomal dominant disease. A mutated copy of the PROC gene is sufficient to cause mild PC deficiency. Severe PC deficiency can occur as a consequence of two altered inherited copies of this gene in each cell of an individual. A thorough review of the literature revealed 14 out of 24 cases of PC deficiency with a positive family history of a thrombotic event. For instance, Wintzen et al. reported a positive family history of VTE and PC deficiency in three siblings of a patient diagnosed with right frontal hemorrhagic infarction [25]. Many studies noted similar observation [2,30,33,37,42]. PC activity was even analyzed in plasma samples from family members of respective patients. The immediate relatives (father, siblings, uncles) of patients screened positive for PC deficiency following an autosomal heterozygous transmission pattern [2,33,42]. Some individuals did not suffer from any thromboembolic event despite having low levels of PC antigen and activity [2,33]. However, death due to coronary artery disease or CVA of unknown cause has also been documented in some families [28,29,35].

PC deficiency can present as multiple thromboembolic events such as DVT, recurrent MI, CVA, and thrombosis of the portal, superior, or inferior mesenteric vein. The previous history of DVT was found significant in studies by Wintzen et al. and Honghong [25,31]. Similarly, some patients were also treated previously for thrombosis of larger veins such as portal, superior, and inferior mesenteric veins [31,33,39]. PC deficiency manifesting as recurrent episodes of CVA or MI has also been highlighted by a number of studies [3,26,28,32,34,37]. Past medical records of some patients revealed atypical symptoms such as generalized tonic-clonic seizures associated with vomiting or a brief period of truncal ataxia and gait disturbance [26,30]. On analysis of other risk factors, a positive relation was found between PC deficiency, and hypertension, smoking, or the use of oral contraceptive pills. Regardless, a few studies showed limited or no association with these risk factors; for example, considering the studies reporting PC deficiency in women, only two females had a significant history of oral contraceptive intake [25,38].

Management

The treatment and management of PC deficiency are mostly based on previously reported cases and experiences. The mainstay of treatment in the majority of cases has been the use of anticoagulants such as heparin, warfarin, aspirin, and clopidogrel [2,24-32]. There exist a paucity of data regarding the management of PC deficiency manifesting as stroke; hence, the aforementioned cases (Table 4) have provided us some clue regarding the failure and success of various therapies. Management summary of cases of stroke due to PC deficiency in the last 10 years is given in Table 5.

**Table 5:** Summary of management of cases reported in the last 10 years LMWH, low molecular weight heparin; Inj, injection; OD, once daily

Study	Initial management	Long term management
Patel et al., 2013 [36]	Heparin	Warfarin
Sultan and Malik, 2013 [26]	Subcutaneous LMWH	Oral anticoagulants
Tomomasa et al., 2013 [40]	Heparin	Warfarin
Honghong, 2015 [31]	25,000 units/day LMWH for 10 days	Warfarin 3 mg/day
Saha et al., 2016 [27]	Inj LMWH	Oral anticoagulants
Li and Qin, 2018 [39]	Heparin	Changing anticoagulant from aspirin to another
Tyagi and Gaur, 2019 [42]	60-mg subcutaneous LMWH for 5 days	3-mg acitrom OD
Tahir et al, 2020 [28]	Aspirin, atorvastatin, clopidogrel, valsartan, bisoprolol, spironolactone, and omeprazole	Anticoagulants, diuretics, beta-blockers, and nitrates

The best management strategy and duration of treatment for most hypercoagulable disorders is unclear. Most physicians use long-term anticoagulation therapy for the prevention of thromboembolic events [44]. Based on the cases in Table 4, cerebral ischemia and infarction secondary to PC deficiency have been successfully managed by initial administration of heparin (mostly low molecular weight heparin) after hospital admission followed by long-term use of anticoagulant (warfarin in most of the cases) [26-28,31,36,39-42].

Several studies have also discussed the management of PC complications such as purpura fulminans. The neonates homozygous for PC deficiency presenting with purpura fulminans or massive venous thrombosis have been successfully managed by starting short-term initial treatment with administration of plasma 8-12 ml/kg every 12 hours. For long-term management, oral anticoagulants or PC replacement have been found beneficial [45]. Administration of human plasma-derived inactivated viral PC concentrate has shown significant improvement in PC activity (up to 94%) in severe cases of purpura fulminans with DIC and alleviated the symptoms [46]. Hepatic transplantation has also been successfully used in resolving the thrombotic condition in a 20-month-old PC deficient child who was previously being managed with fresh frozen plasma and warfarin. The child was administered 3 mg/kg heparin intravenous bolus immediately post-operation, and maintenance therapy was started with a once-daily dose of 40 mg/kg aspirin and a twice-daily dose of 1 mg/kg dipyridamole [47].

In 2018, the American Society of Hematology (ASH) recommended the use of PC concentrate over anticoagulants (due to decreased risk of bleeding) for the treatment of purpura fulminans in homozygous PC deficiency; however, lack of cost-effectiveness may limit PC concentrate use. PC concentrate with anticoagulants is superior to PC concentrate alone as it reduces the intensity of anticoagulants required and thus reduces the risk of bleeding. ASH guidelines have also recommended liver transplant in PC deficient patients, but it has its own associated acute and chronic risks [48].

## Conclusions

PC deficiency is a well-established cause for the development of stroke, particularly in young adults. Although VTE has been widely discussed, few studies have also shown the increased risk of ATE with this deficiency, pointing toward the need for further cohort studies. Family history and a history of thrombotic events (such as DVT, recurrent MI or CVA, and thrombosis of the portal, superior, or inferior mesenteric vein) in patients of stroke without a clear underlying pathology should always be considered significant for timely diagnosis and effective management. The mainstay of treatment in the majority of cases has been the use of anticoagulants such as heparin, warfarin, aspirin, and clopidogrel. Counselling of the patients regarding the probable outcomes and risk factors that can aggravate the condition can help with medication compliance and reduction in recurrence rate.

## References

[1] (2020). Protein C deficiency. https://emedicine.medscape.com/article/205470-overview.

[2] Kohler J, Kasper J, Witt I, Von Reutern GM (1990). Ischemic stroke due to protein C deficiency. Stroke.

[3] Arkel YS, Ku DH, Gibson D, Lam X (2020). Ischemic stroke in a young patient with protein C deficiency and prothrombin gene mutation G20210A. Blood Coagul Fibrinolysis.

[4] Chiasakul T, De Jesus E, Tong J (2020). Inherited thrombophilia and the risk of arterial ischemic stroke: a systematic review and meta‐analysis. J Am Heart Assoc.

[5] Palta S, Saroa R, Palta A (2014). Overview of the coagulation system. Indian J Anaesth.

[6] Dahlbäck B, Villoutreix BO (2005). The anticoagulant protein C pathway. FEBS Lett.

[7] Xue M, Jackson CJ (2015). Novel functions of the anticoagulant activated protein C in maintaining skin barrier integrity to impact on skin disease. Pathobiology.

[8] Shahzad K, Kohli S, Isermann B (2019). Cell biology of activated protein C. Curr Opin Hematol.

[9] Lensen RP, Rosendaal FR, Koster T (1996). Apparent different thrombotic tendency in patients with factor V Leiden and protein C deficiency due to selection of patients. Blood.

[10] Knoebl PN (2008). Severe congenital protein C deficiency: the use of protein C concentrates (human) as replacement therapy for life-threatening blood-clotting complications. Biologics.

[11] Pescatore SL (2001). Clinical management of protein C deficiency. Expert Opin Pharmacother.

[12] Hart RG, Kanter MC (1990). Hematologic disorders and ischemic stroke. A selective review. Stroke.

[13] Martinez HR, Rangel-Guerra RA, Marfil LJ (1993). Ischemic stroke due to deficiency of coagulation inhibitors. Report of 10 young adults. Stroke.

[14] Camerlingo M, Finazzi G, Casto L, Laffranchi C, Barbui T, Mamoli A (1991). Inherited protein C deficiency and nonhemorrhagic arterial stroke in young adults. Neurology.

[15] Zakai NA, Judd SE, Kissela B, Howard G, Safford MM, Cushman M (2018). Factor VIII, protein C and cardiovascular disease risk: the reasons for geographic and racial differences in stroke study (REGARDS). Thromb Haemost.

[16] Hankey GJ, Eikelboom JW, van Bockxmeer FM, Lofthouse E, Staples N, Baker RI (2001). Inherited thrombophilia in ischemic stroke and its pathogenic subtypes. Stroke.

[17] 17] Houghton J (2020). Protein C defiency: a review for clinicians by Houghton J. https://research.wsulibs.wsu.edu:8443/xmlui/handle/2376/3561.

[18] Mateo J, Oliver A, Borrell M, Sala N, Fontcuberta J (1997). Laboratory evaluation and clinical characteristics of 2,132 consecutive unselected patients with venous thromboembolism-results of the Spanish multicentric study on thrombophilia (EMET-study). Thromb Haemost.

[19] Boekholdt SM, Kramer MH (2007). Arterial thrombosis and the role of thrombophilia. Semin Thromb Hemost.

[20] Lefebvre P, Lierneux B, Lenaerts L (1998). Cerebral venous thrombosis and procoagulant factors: a case study. Angiology.

[21] Hotoleanu C (2017). Genetic risk factors in venous thromboembolism. Adv Exp Med Biol.

[22] Mahmoodi BK, Brouwer JL, Veeger NJ, van der Meer J (2008). Hereditary deficiency of protein C or protein S confers increased risk of arterial thromboembolic events at a young age: results from a large family cohort study. Circulation.

[23] Adams HP, Kappelle LJ, Biller J, Gordon DL, Love BB, Gomez F, Heffner M (1995). Ischemic stroke in young adults: experience in 329 patients enrolled in the Iowa registry of stroke in young adults. Arch Neurol.

[24] Ranawaka UK, Alibhoy AT, Wijesekera JC (2001). Three young patients with unusual causes of stroke. The Ceylon Med J.

[25] Wintzen AR, Broekmans AW, Bertina RM (1985). Cerebral haemorrhagic infarction in young patients with hereditary protein C deficiency: evidence for" spontaneous" cerebral venous thrombosis. Br Med J (Clin Res Ed).

[26] Sultan A, Malik IH (2013). Recurrent cerebral infarctions in a young patient: combined protein C and S deficiencies. J Coll Physicians Surg Pak.

[27] Saha DK, Ahsan AA, Fatema K (2016). Ischemic stroke due to protein C deficiency. Bangladesh Crit Care J.

[28] Tahir F, Majid Z, Bin Arif T, Ahmed J (2020). Cerebral infarction followed by myocardial infarction in a young adult with protein C and S deficiency. Cureus.

[29] Yang FC, Hsu CH, Lin JC, Chen CY, Lee JT (2008). Inherited protein C deficiency with acute ischemic stroke in a young adult: a case report. Blood Coagul Fibrinolysis.

[30] Kato H, Shirahama M, Ohmori K, Sunaga T (1995). Cerebral infarction in a young adult associated with protein C deficiency: a case report. Angiology.

[31] Honghong L (2015). Two cases of recurrent vascular events due to protein C deficiency. J Clin Toxicol.

[32] Tiong IY, Alkotob ML, Ghaffari S (2003). Protein C deficiency manifesting as an acute myocardial infarction and ischaemic stroke. Heart.

[33] Deguchi K, Tsukada T, Iwasaki E, Wada H, Murashima S, Miyazaki M, Shirakawa S (1992). Late-onset homozygous protein C deficiency manifesting cerebral infarction as the first symptom at age 27. Intern Med.

[34] Kikuta KI, Miyamoto S, Kataoka H, Yamada K, Takagi Y, Nozaki K, Hashimoto N (2005). An adult case of moyamoya syndrome that developed dural sinus thrombosis associated with protein C deficiency: case report and literature review. Surg Neurol.

[35] Matsushita K, Kuriyama Y, Sawada T, Uchida K (1992). Cerebral infarction associated with protein C deficiency. Stroke.

[36] Patel ML, Sachan R, Seth G (2020). Combined deficiency of proteins C and S: ischaemic stroke in young individuals. BMJ Case Rep.

[37] Weir NU, Snowden JA, Greaves M, Davies‐Jones GA (1995). Livedo reticularis associated with hereditary protein C deficiency and recurrent thromboembolism. Br J Dermatol.

[38] Celebisoy N, Sagduyu A, Atac C (2005). Alexia without agraphia following cerebral venous thrombosis associated with protein C and protein S deficiency. Clin Neurol Neurosurg.

[39] Li P, Qin C (2018). Recurrent cerebellar infarction associated with hereditary heterozygous protein C deficiency in a 35‑year‑old woman: a case report and genetic study on the pedigree. Exp Ther Med.

[40] Tomomasa R, Yamashiro K, Tanaka R, Hattori N (2013). Cerebral infarction in an HIV-infected patient with combined protein S and C deficiency and a patent foramen ovale. J Stroke Cerebrovasc Dis.

[41] Kazui S, Kuriyama Y, Sakata T, Hiroki M, Miyashita K, Sawada T (1993). Accelerated brain infarction in hypertension complicated by hereditary heterozygous protein C deficiency. Stroke.

[42] Tyagi S, Gaur N (2019). Heterozygous protein-c deficiency presenting as stroke due to cerebral venous sinus thrombosis: a case report. IJCR.

[43] Chiu YN, Wang TG, Chang CW (2009). Early-onset stroke in a patient with nasopharyngeal cancer associated with protein C deficiency. J Formos Med Assoc.

[44] Mackey Mackey, J J (2014). Evaluation and management of stroke in young adults. Continuum (Minneap Minn).

[45] Marlar RA, Montgomery RR, Broekmans AW (1989). Diagnosis and treatment of homozygous protein C deficiency. J Pediatr.

[46] Gerson WT, Dickerman JD, Bovill EG, Golden E (1993). Severe acquired protein C deficiency in purpura fulminans associated with disseminated intravascular coagulation: treatment with protein C concentrate. Pediatrics.

[47] Casella J, Bontempo F, Markel H, Lewis J, Zitelli B, Starzl T (1988). Successful treatment of homozygous protein C deficiency by hepatic transplantation. The Lancet.

[48] Monagle P, Cuello CA, Augustine C (2018). American society of hematology 2018 guidelines for management of venous thromboembolism: treatment of pediatric venous thromboembolism. Blood Adv.

